# Comparative immunoproteomics of *Campylobacter jejuni* in acute campylobacteriosis and asymptomatic individuals

**DOI:** 10.3389/fcimb.2026.1800725

**Published:** 2026-05-28

**Authors:** Fernando A. Gómez, Isabel Briceño, Arturo Levican

**Affiliations:** 1 Laboratorio de Genética e Inmunología Molecular, Instituto de Biología, Facultad de Ciencias, Pontificia Universidad Católica de Valparaíso, Valparaíso, Chile; 2 Laboratorio Clínico, Hospital Naval Almirante Nef, Viña del Mar, Chile; 3 Escuela de Tecnología Médica, Facultad de Ciencias, Pontificia Universidad Católica de Valparaíso, Valparaíso, Chile; 4 Centro de Investigación Interdisciplinaria en Biomedicina, Biotecnología y Bienestar (CID3B). Pontificia Universidad Católica de Valparaíso, Valparaíso, Chile

**Keywords:** acute infection, bioinformatic analysis, *Campylobacter jejuni*, immunoproteomic analysis, mass spectrometry, nano liquid chromatography

## Abstract

*Campylobacter jejuni* is a leading cause of bacterial gastroenteritis worldwide; however, the molecular determinants associated with symptomatic infection remain incompletely understood. In this study, an immunoproteomic approach was applied to characterize the antigenic landscape of *C. jejuni* during acute campylobacteriosis and to identify infection-associated proteins targeted by the human humoral immune response. Sera from patients with laboratory-confirmed acute campylobacteriosis (Group A) and asymptomatic individuals without recent gastrointestinal disease (Group B) were used to immunocapture bacterial proteins, followed by nano–liquid chromatography–tandem mass spectrometry analysis. A total of 418 and 517 immunoreactive proteins were identified in Groups A and B, respectively, with a large shared core repertoire, indicating extensive background immune recognition of conserved bacterial components. Functional annotation revealed that metabolic enzymes and ribosomal proteins dominated the immunoproteome in both groups. However, semi-quantitative emPAI analysis identified selective differences associated with acute infection. Group A sera preferentially recognized proteins involved in stress response, motility, secretion, and virulence-related functions, whereas Group B sera predominantly recognized housekeeping proteins. Bioinformatic prediction and network analyses identified immunoreactive proteins with features compatible with secretion-associated virulence factors, including proteins related to the flagellar export apparatus and the cytolethal distending toxin complex. Additionally, several immunoreactive proteins carried C-terminal acidic E-Block–like motifs previously described in Type IV secretion system effectors from other bacterial pathogens. Although the present approach does not directly demonstrate secretion, surface exposure, or translocation of the predicted proteins, these findings provide insight into infection-associated immune recognition patterns and identify candidate virulence-associated antigens that warrant future experimental validation.

## Introduction

*Campylobacter jejuni* is recognized as one of the leading bacterial causes of acute gastroenteritis worldwide and represents a major public health concern in both industrialized and developing countries (Young et al., 2007; Bolton, 2015). The incidence of campylobacteriosis has increased steadily over recent decades, surpassing that of other foodborne bacterial pathogens in many regions, particularly in Europe and North America (European Food Safety AuthorityEuropean Centre for Disease Prevention and Control, 2021; Kaakoush et al., 2015). Infection is commonly associated with acute diarrhea, abdominal pain, fever, and vomiting; however, in a subset of patients, *C. jejuni* infection may lead to severe post-infectious sequelae such as Guillain–Barré syndrome, Miller Fisher syndrome, and reactive arthritis, highlighting its clinical relevance beyond self-limiting gastroenteritis (Nachamkin, 1997; Godschalk et al., 2004).

The pathogenicity of *C. jejuni* is multifactorial and depends on the coordinated action of motility, adhesion, invasion, toxin production, and resistance to host-derived stress conditions (Young et al., 2007). Motility, mediated by a bipolar flagellum, is essential for intestinal colonization and traversal of the mucus layer, while adhesion and invasion are facilitated by surface-exposed proteins such as CadF and PEB1 (Konkel et al., 1997; Monteville et al., 2003). In addition, *C. jejuni* produces the cytolethal distending toxin (CDT), a tripartite toxin composed of CdtA, CdtB, and CdtC, whose CdtB subunit exhibits DNase-like activity that induces DNA damage, cell cycle arrest, and apoptosis in host cells (Lara-Tejero and Galán, 2001; Ge et al., 2008). CDT is considered one of the most important virulence determinants contributing to epithelial damage and inflammation during infection.

Unlike many Gram-negative pathogens, *C. jejuni* lacks a canonical injectisome-type Type III secretion system (T3SS). Instead, it exploits the flagellar export apparatus to secrete virulence-associated proteins, including the Campylobacter invasion antigens (Cia proteins), directly into host epithelial cells (Konkel et al., 1999; Neal-McKinney and Konkel, 2012). This non-canonical secretion strategy underscores the functional versatility of the flagellum and its central role in host–pathogen interactions. Flagellar components and associated proteins have also been implicated in immune recognition, further supporting their relevance during infection (Barrero-Tobón and Hendrixson, 2012; Barrero-Tobón and Hendrixson, 2014).

In parallel, multidrug efflux systems play a crucial role in the survival and pathogenicity of *C. jejuni*. The CmeABC efflux pump is the best-characterized resistance–nodulation–division (RND) family transporter in this species and is involved not only in antimicrobial resistance but also in bile tolerance, intestinal colonization, and adaptation to the host environment (Lin et al., 2002; Luangtongkum et al., 2009; Guo et al., 2010). These findings highlight the intimate link between antimicrobial resistance mechanisms and virulence traits in *C. jejuni*, reinforcing the concept that factors traditionally associated with resistance may also contribute to pathogenic fitness.

Although *C. jejuni* has historically not been considered a classical intracellular pathogen, increasing evidence suggests that it can transiently survive within host cells and manipulate host processes through secreted effectors (Young et al., 2007; Neal-McKinney and Konkel, 2012). In this context, secretion systems beyond the flagellar T3SS-like pathway have gained attention. Type IV secretion systems (T4SSs), well characterized in other bacterial pathogens such as *Legionella pneumophila* and *Coxiella burnetii*, mediate the translocation of effector proteins into host cells using C-terminal secretion signals, including acidic E-Block motifs (Nagai et al., 2005; Burstein et al., 2009). The potential existence of T4SS-like mechanisms or substrates in *C. jejuni* remains poorly explored and represents an important knowledge gap.

While genomic and conventional proteomic studies have provided extensive information on the repertoire of virulence-associated genes and proteins in *C. jejuni* (Cordwell et al., 2008; Parker et al., 2016), these approaches do not necessarily reflect which bacterial proteins are actually expressed, secreted, or immunologically relevant during human infection. Importantly, the presence of a gene or protein does not imply immune recognition, particularly in the context of acute disease versus asymptomatic exposure.

Immunoproteomics offers a powerful complementary strategy to address this limitation by identifying bacterial proteins recognized by host antibodies, thereby providing insight into antigens that are expressed and exposed to the immune system *in vivo* (Cordwell et al., 2008). Comparative immunoproteomic analyses using sera from symptomatic and asymptomatic individuals can reveal subtle but biologically meaningful differences in immune recognition, contributing to the identification of virulence-associated antigens, biomarkers of active infection, and potential vaccine candidates.

In this study, we applied an immunoproteomic approach combined with functional annotation, protein–protein interaction network analysis, and secretion system prediction to characterize *C. jejuni* proteins recognized by sera from patients with acute campylobacteriosis and asymptomatic individuals. By integrating quantitative and bioinformatic analyses, we aimed to identify virulence-associated and secretion-related proteins that may contribute to the pathogenesis of acute infection and to expand current knowledge of host–pathogen interactions in *C. jejuni*.

## Materials and methods

### Bacterial strains and sera samples

Representative strains of *C. jejuni* (PUCV-1, PUCV-2 and PUCV-3) were previously selected (Levican et al., 2019) as they were considered representatives of the diversity present among patients with acute diarrhea assisted at the Hospital Naval Almirante Nef, Viña del Mar, Chile, on the basis of flaA-RFLP, corresponding to distinct cgMLST profiles (50, 353 and 475, respectively) and clonal complexes (21, 353 and 48, respectively). In addition, the type strain of *C. jejuni* ATCC 33560^T^ was included. All strains were maintained at -80 °C in 15% glycerol, and were recovered on Trypticase soy agar (Becton Dickinson, USA) supplemented with 5% sheep blood incubated 48 hours at 42 °C under microaerobic conditions. However, as recommended to obtain a better expression of virulence related proteins (Scott and Cordwell, 2009), the isolates were grown on Trypticase soy agar (Becton Dickinson, USA) supplemented with 5% sheep blood and 1% deoxycholate (Sigma, USA) and incubated for 48 hours at 37 °C under microaerobic conditions.

Sera samples were obtained from blood samples of patients assisted at the Hospital Naval Almirante Nef, Viña del Mar, Chile, between November 2018 and October 2019, after requesting their informed consent. Two groups of sera were formed by including blood samples from participants with and without acute campylobacteriosis, respectively. Campylobacteriosis was defined as acute diarrhea, i.e. more than 3 loose stools in 24 hrs. together with a positive culture for *C. jejuni* from fecal samples, and in this group of participants the samples were obtained during the acute symptomatic period. On the other hand, participants without acute campylobacteriosis were defined as they did not present this diarrheic syndrome at least 6 months before the sampling date.

To ensure serological discrimination between groups, all sera were tested using ELISA Anti-*Campylobacter jejuni* IgG and ELISA Anti-*Campylobacter jejuni* IgA kits (Euroimmun, Germany). Only sera positive for both IgG and IgA were included in Group A, whereas only double-negative sera were included in Group B; samples with discordant results were excluded. Sera from each group were pooled, aliquoted, and stored at −80 °C until use. The use of pooled sera was intended to reduce individual variability and to obtain a representative immunoreactive profile for each study group. However, this approach does not allow assessment of inter-individual variation or statistical comparison between individual responses.

### Proteomic analysis and immunocapture

Proteomic analyses were performed by nano–liquid chromatography coupled to tandem mass spectrometry (nLC–MS/MS) at Instituto Melisa (Concepción, Chile). Immunoglobulins from pooled serum samples of Groups A and B were purified using the PureProteome kit (Merck Millipore, USA) according to the manufacturer’s instructions and immobilized on protein A–conjugated magnetic beads using the Protein A Magnetic Beads kit (Merck Millipore, USA). Successful immunoglobulin immobilization was verified by 10% SDS-PAGE.

In parallel, bacterial proteins were extracted independently from *C. jejuni* PUCV-1, PUCV-2, PUCV-3, and ATCC 33560^T^ strains. Protein extracts were precipitated, concentrated, and pooled in equal proportions from each strain. The pooled protein preparation was digested with sequencing-grade trypsin (Promega, USA) at a protease-to-protein ratio of 1:50 (w/w). Peptide clean-up was performed using Sep-Pak C18 Spin Columns (Waters, USA) following the manufacturer’s recommendations. Purified peptides were dried overnight at 10 °C using a rotary concentrator (1000 rpm).

### Immunocapture of peptides

Tryptic peptides were incubated with immobilized immunoglobulins for 12 h to allow immunocapture of antigenic peptides. After incubation, unbound peptides were removed by washing with phosphate-buffered saline (PBS). Bound peptides were eluted by acidification (1% formic acid), followed by precipitation of residual immunoglobulins. To ensure complete removal of immunoglobulins, eluates were filtered using Vivaspin 10 kDa centrifugal devices (Sartorius, Germany).

### Mass spectrometry analysis

A total of 200 ng of peptides were injected into an Evosep One system (Evosep Biosystems) coupled to a timsTOF Pro mass spectrometer (Bruker Daltonics, Germany) equipped with trapped ion mobility spectrometry (TIMS). Peptide separation was performed using a PepSep Eight column (8 cm × 150 μm ID, 1.5 μm C18; Bruker Daltonics).

Liquid chromatography was conducted using the 60 samples per day (SPD) mode for culture and concentrated samples, and the 100 SPD mode for purified samples. The gradient ranged from 2% to 35% buffer B (0.1% formic acid in acetonitrile). Data acquisition was performed using TimsControl 2.0 (Bruker Daltonics) with 10 PASEF cycles, a mass range of 100–1700 m/z, capillary voltage of 1500 V, capillary temperature of 180 °C, TOF frequency of 10 kHz, and a resolution of 50,000 FWHM.

### Protein identification and annotation

Raw mass spectrometry data were processed using MSFragger version 3.7 (Kong et al., 2017) implemented in FragPipe v19.1, using default workflows on a high-performance computing server (48 cores, 512 GB RAM). Precursor mass tolerance was set to −20 to +20 ppm and fragment mass tolerance to 20 ppm. Trypsin was specified as the proteolytic enzyme with up to two missed cleavages. Carbamidomethylation of cysteine was set as a fixed modification, while methionine oxidation and N-terminal acetylation were set as variable modifications.

Protein identification was performed against a customized database comprising predicted proteins from *C. jejuni* PUCV-1, PUCV-2, and PUCV-3 genomes (BioProject PRJNA531695) and *C. jejuni* ATCC 33560^T^ (accession GCA_000254515). False discovery rate (FDR) was controlled at <1% using a decoy database. A contaminant database was included to account for common mass spectrometry contaminants.

Functional annotation was performed using DIAMOND BLASTp (Buchfink et al., 2015) against the Virulence Factor Database (Liu et al., 2019) and the Comprehensive Antibiotic Resistance Database (Alcock et al., 2020), applying an e-value threshold of 1e−10 and retaining the best hit based on bit score.

### Quantitative estimation of protein abundance

Relative protein abundance was estimated using the exponentially modified Protein Abundance Index (emPAI) as described by Ishihama et al. (2005). emPAI values were calculated based on the ratio between the number of observed peptides and the number of theoretically observable peptides for each protein and were used as a label-free proxy for protein abundance.

### Functional annotation and subsystem classification

All identified proteins were annotated against UniProtKB/Swiss-Prot and NCBI RefSeq databases using DIAMOND BLASTp. Proteins were subsequently classified into RAST-like functional subsystems, including metabolism, protein synthesis, stress response, DNA/RNA metabolism, cell envelope, membrane transport and secretion, motility and chemotaxis, virulence/defense, and other functions. The complete subsystem mapping is provided in **Supplementary Table 1**.

### Prediction of Type III secretion system effectors

Putative T3SS effectors were predicted using both Effectidor (Wagner et al., 2022) and the EffectiveT3 module from EffectiveDB (Eichinger et al., 2016). Proteins with Effectidor scores ≥0.5 were considered candidate T3SS effectors, and those scoring ≥0.8 were classified as high-confidence candidates. Predictions were further supported by criteria of N-terminal secretion-associated features, including low-complexity regions and serine/threonine enrichment, following established T3SS signal criteria (Galán and Wolf-Watz, 2006).

### Prediction of Type IV secretion system effectors

Candidate Type IV secretion system effectors were identified using S4TE 2.0 (Noroy et al., 2019). Proteins with a global S4TE score ≥70 or ranking within the top 10% were retained. Predictions were cross-referenced against SecReT4 (Bi et al., 2013), a curated database of T4SS components and effectors. Proteins supported by both S4TE and SecReT4, or with S4TE scores ≥90 alone, were classified as high-confidence T4SS effector candidates.

### Protein–protein interaction networks and enrichment analysis

Protein–protein interaction networks were constructed using STRING v12 (Szklarczyk et al., 2023), using *C. jejuni* as the reference organism (wild type). Medium-confidence (0.4) and high-confidence (0.7) interaction thresholds were applied for global and focused analyses, respectively. Functional enrichment analyses for Gene Ontology biological processes, KEGG pathways, and InterPro domains were performed with Benjamini–Hochberg FDR correction, retaining terms with q < 0.05.

### Prioritization of infection-associated proteins

Proteins were prioritized as potential infection-stage markers based on three criteria: (i) presence and/or increased emPAI values in Group A sera, (ii) predicted effector status (T3SS and/or T4SS), and (iii) clustering within STRING interaction networks associated with known virulence modules, including the CdtABC toxin complex, CmeABC efflux system, flagellar export apparatus, adhesin complexes, and periplasmic folding pathways. Proteins fulfilling at least two criteria were considered high-priority candidates for further validation.

## Results

### Study population and serological classification

A total of 563 stool samples were screened during the study period, yielding 11 confirmed *C. jejuni* isolates (1.96%). Six patients with laboratory-confirmed acute campylobacteriosis and positive anti-*C. jejuni* IgA/IgG responses, which consent to participate in the study, were included as Group A, representing individuals with active diarrheal disease. Sera from five asymptomatic volunteers with no gastrointestinal symptoms in the preceding six months and negative serology were used as controls. This classification allowed a clear distinction between acute infection–associated immune responses and background serological recognition. It should be noted that sera were analyzed in pooled format, and therefore the results reflect group-level immunoreactivity patterns rather than individual-specific responses.

### Global immunoproteomic landscape and core antigenic repertoire

**Figure 1:**
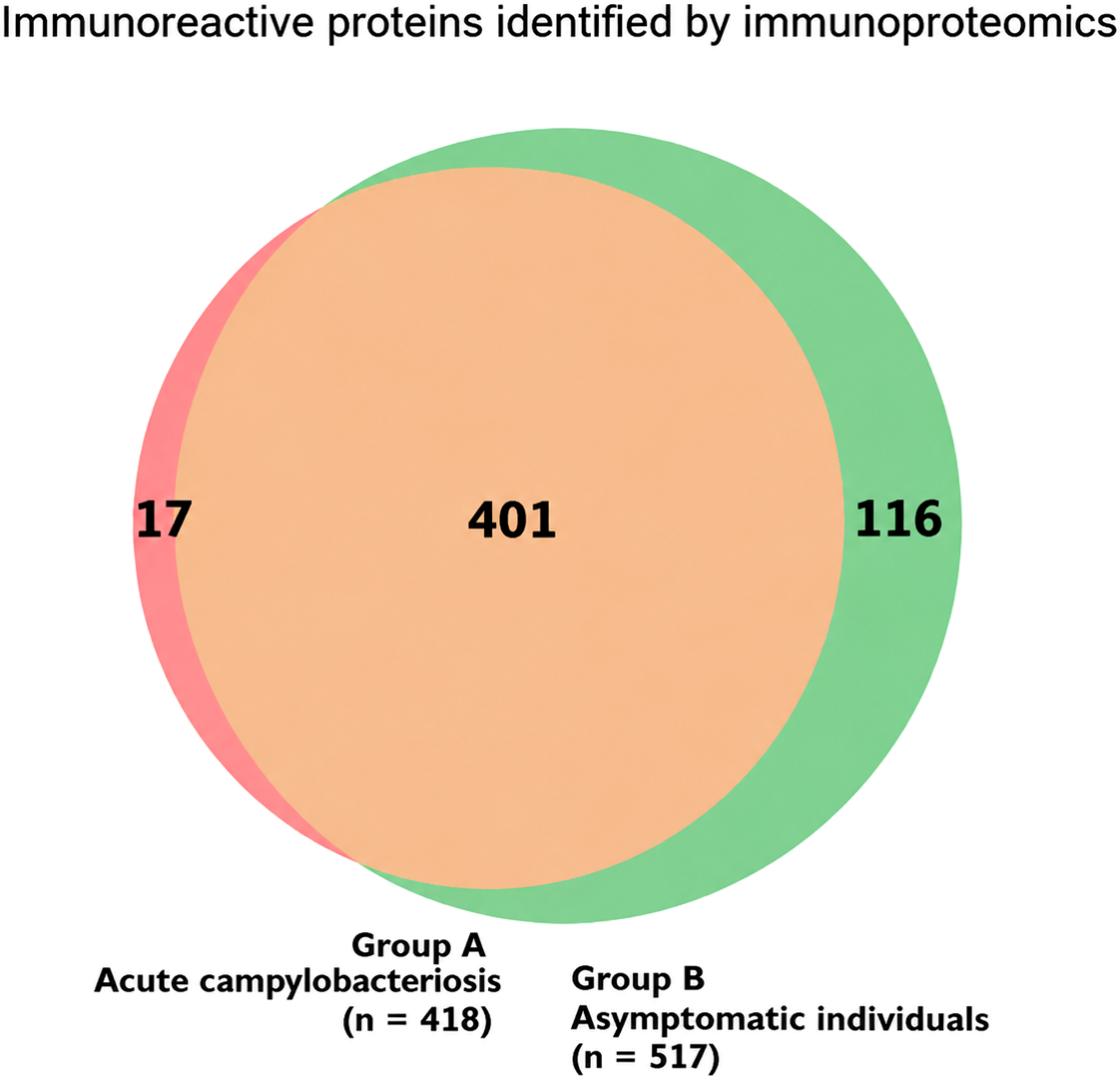
Global Venn diagram of *C. jejuni* immunoreactive proteins identified using sera from patients with acute campylobacteriosis (Group A) and asymptomatic individuals (Group B). A total of 418 and 517 proteins were detected in Groups A and B, respectively, with 401 proteins shared between both groups, 17 proteins exclusively detected in Group A, and 116 exclusively detected in Group B.

Immunocapture coupled to nLC–MS/MS identified 418 immunoreactive proteins in Group A sera and 517 proteins in asymptomatic controls, revealing a broad antigenic landscape recognized by the human immune system. A substantial shared core of 401 proteins was detected in both groups, indicating that most immunoreactive proteins correspond to conserved bacterial components commonly recognized regardless of clinical status (**Figure 1**). Despite this large overlap, distinct subsets of group-specific proteins were identified. Group A sera uniquely recognized 17 proteins, whereas 116 proteins were exclusively detected in asymptomatic controls, highlighting differential immune recognition patterns associated with acute infection versus asymptomatic exposure. These findings suggest that active campylobacteriosis is characterized not by a completely distinct antigenic repertoire, but by selective recognition of a restricted subset of proteins within a largely shared background. This substantial overlap likely reflects a strong background of immune recognition of conserved and highly abundant bacterial proteins, which may limit the degree of separation between clinical groups.

### Functional subsystem distribution reveals subtle but consistent differences between groups

**Figure 2:**
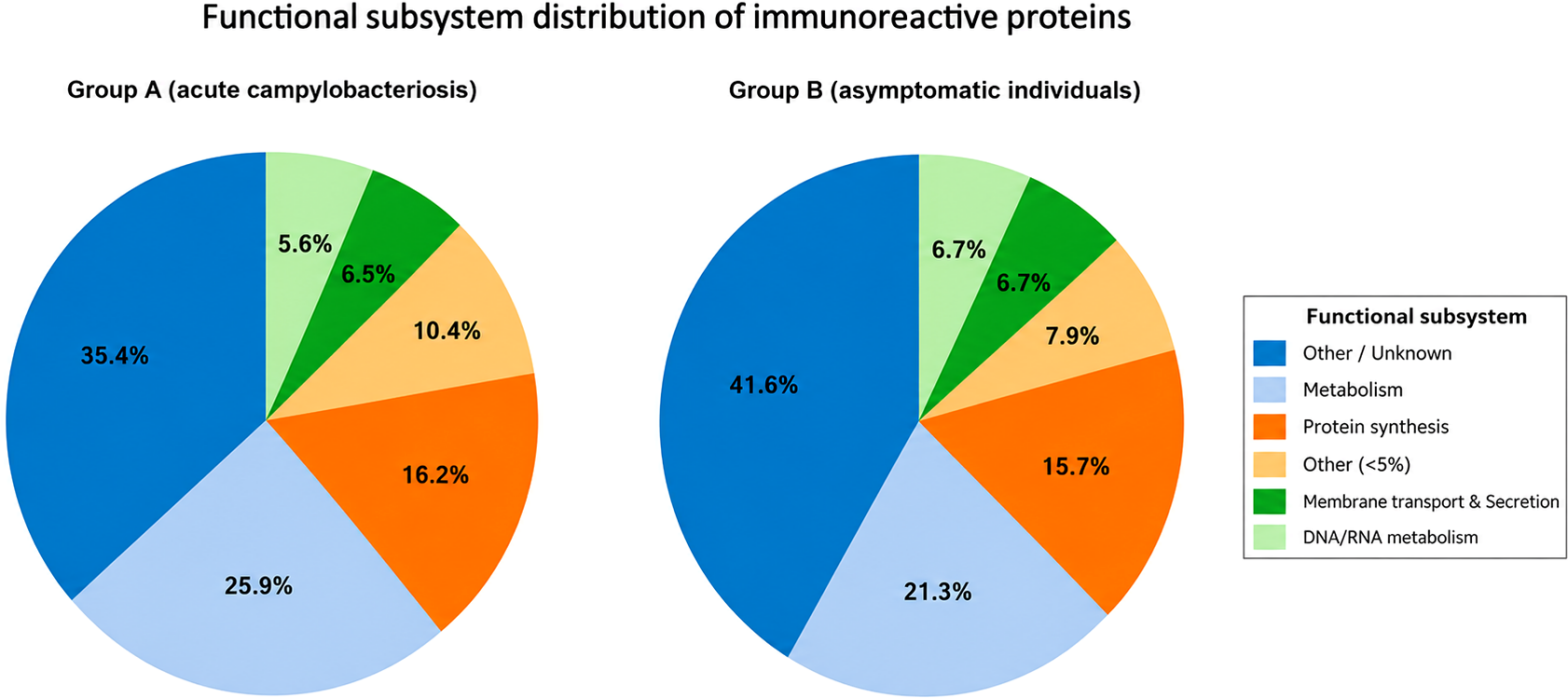
Functional subsystem distribution of immunoreactive proteins identified in sera from patients with acute campylobacteriosis (Group A) and (B) asymptomatic individuals (Group B). Proteins were classified into compact RAST-like functional subsystems as reported in **Supplementary Table 1**. Minor categories representing less than 5% of the total were grouped as “Other (<5%).”.

Functional annotation and classification of immunoreactive proteins into compact RAST-like subsystems showed that metabolism, protein synthesis, and other poorly characterized proteins accounted for the majority of antigens in both groups (**Supplementary Table 1**; **Figure 2**). This distribution reflects the immunodominance of abundant and conserved bacterial proteins, such as metabolic enzymes and ribosomal components, including ribosomal proteins, metabolic enzymes, and molecular chaperones, which are commonly detected in immunoproteomic studies due to their high abundance and broad immunogenicity. Comparative analysis revealed quantitative differences in several functional categories. Proteins involved in membrane transport and secretion were more frequently detected in asymptomatic controls, whereas Group A sera showed a relative enrichment in proteins associated with stress response and virulence/defense. Although these categories represented a small fraction of the total proteome, their preferential detection in Group A suggests an infection-driven modulation of antigen recognition during acute disease. Proteins related to motility and chemotaxis and cell envelope/periplasmic functions were consistently identified in both groups, underscoring the importance of surface-exposed and motility-associated structures in immune recognition.

### Virulence-associated proteins and secretion-related factors are preferentially recognized during acute infection

Targeted bioinformatic screening against virulence and resistance databases identified multiple proteins previously associated with host interaction or bacterial fitness (**Table 1**). Among these, the cytolethal distending toxin subunit CdtB was detected and predicted by *in silico* tools to present features compatible with Type III secretion. Components of the CmeABC multidrug efflux system, including CmeC, were also identified and clustered with CmeA, CmeB, and the transcriptional regulator CmeR in STRING-based interaction analysis, suggesting their coordinated functional association. These proteins were detected in Group A sera, indicating immune recognition during acute infection, although this approach does not allow conclusions regarding their expression levels, localization, or direct involvement in pathogenesis. Flagellar-associated proteins such as FlgE were also identified and predicted as potential Type III secretion candidates, consistent with the known association of the flagellar apparatus with protein export in *C. jejuni*. In addition, selected proteins carrying secretion-related sequence features, including C-terminal E-Block–like motifs previously associated with Type IV secretion system substrates in other bacterial pathogens, were identified based on prediction tools. However, no genomic or functional evidence of a Type IV secretion system is currently available in *C. jejuni*, and these observations should therefore be interpreted cautiously.

**Table 1:** Virulence-associated proteins identified by immunoproteomics.

Protein ID	Protein name/annotation	Functional category	Group A detected	Group B detected	Evidence (VFDB/CARD/STRING/T3SS/T4SS)	Notes
CdtB	Cytolethal distending toxin subunit B	Virulence/Defense	Yes	Yes	VFDB; STRING (CDT module); T3SS-like export	Central toxin component
CiaB	Campylobacter invasion antigen B	Virulence/Defense	Yes	No	VFDB; T3SS (Effectidor/EffectiveT3)	Invasion-associated
CadF	Fibronectin-binding protein	Adhesion	Yes	Yes	VFDB; STRING (adhesin cluster)	Host cell binding
PEB1a/PEB3	Periplasmic binding proteins	Adhesion/Transport	Yes	Yes	VFDB; STRING	Colonization
CmeA	Efflux system component	Resistance/Virulence	Yes	Yes	CARD; STRING (CmeABC)	Multidrug efflux
CmeB	Efflux pump	Resistance/Virulence	Yes	Yes	CARD; STRING (CmeABC)	Multidrug efflux
CmeC	Outer membrane component	Resistance/Virulence	Yes	Yes	CARD; STRING (CmeABC)	Multidrug efflux
FlgE	Flagellar hook protein	Motility	Yes	Yes	VFDB; T3SS-like export	Flagellar secretion
MotA	Flagellar motor protein	Motility	Yes	Yes	VFDB; STRING	Motility/energy
Pal (TolB)	Peptidoglycan-associated lipoprotein	Cell envelope	Yes	Yes	T4SS (S4TE/SecReT4); E-Block	Envelope stability

### Prediction of type III secretion system effectors

Using the EffectiveT3 server and the Effectidor platform, a total of 32 proteins were predicted as potential Type III secretion system (T3SS) effector candidates in *C. jejuni* (**Supplementary Table 2**). Several of these proteins displayed high prediction scores (≥0.99); however, these values should be interpreted as indicative rather than definitive evidence of secretion. Among the predicted candidates, proteins with annotated functions included ribonuclease J (TWO77279.1), the outer membrane efflux component CmeC (TWO77761.1), and the cytolethal distending toxin subunit CdtB (TWO77628.1). In addition, multiple hypotheticals or non-classically secreted proteins, including cytoskeletal-like and radical SAM/SPASM domain-containing proteins, were also identified. While these predictions may point to proteins with features compatible with T3SS-associated export, no experimental validation of secretion or translocation was performed in this study, and the possibility of false-positive predictions cannot be excluded. The identification of CdtB (TWO77628.1) among the predicted T3SS candidate proteins is noteworthy, given its well-established role within the cytolethal distending toxin complex. However, its classification here is based on in silico prediction, and its potential export through the flagellar secretion apparatus cannot be inferred from the present data alone.

### STRING-based functional interaction network of T3SS-associated proteins

To contextualize the predicted T3SS effectors, a protein–protein interaction network analysis was performed using STRING. This analysis revealed the formation of distinct functional clusters, providing biological coherence to the computational predictions (**Supplementary Table 2**). The CdtB protein (TWO77628.1) formed a well-defined interaction module with its cognate subunits CdtA and CdtC, constituting the canonical CDT toxin complex. This cluster was strongly associated with processes related to DNA damage and cell cycle modulation, confirming the central role of CDT in *C. jejuni* virulence. A second prominent cluster was centered on CmeC (TWO77761.1), which showed direct interactions with CmeA, CmeB, and the transcriptional regulator CmeR, corresponding to the CmeABC multidrug efflux system. This network highlights the functional integration of CmeC within resistance, persistence, and host colonization pathways, linking antimicrobial resistance mechanisms with virulence-associated traits.

Additional nodes included FtsH (TWO78303.1), a membrane-associated zinc metalloprotease involved in protein quality control, and ribonuclease J (TWO77279.1), which clustered with ribonucleases and ribosomal-associated proteins. Although these proteins are not classical effectors, their network connectivity suggests indirect roles in regulating membrane protein homeostasis and post-transcriptional control of virulence-associated genes.

### Identification of putative type IV secretion system) effectors carrying E-Block motifs

To explore the potential presence of proteins with features compatible with Type IV secretion system (T4SS) substrates, the Secret4 and S4TE servers were employed to identify candidate proteins *in C. jejuni*. This analysis yielded eight proteins showing sequence similarity to previously described T4SS-associated proteins from other bacterial pathogens (**Table 2**). Examination of their C-terminal regions revealed the presence of acidic E-Block–like motifs, characterized by glutamate-rich sequences within the last ~30 amino acids. Similar motifs have been reported in T4SS substrates of intracellular pathogens such as *Legionella pneumophila*, *Coxiella burnetii*, and *Piscirickettsia salmonis.* Among the proteins identified were Pal (TWO77661.1), CdtB (TWO77628.1), CiaB (TWO78491.1), ankyrin repeat–containing proteins (TWO75222.1), and several hypothetical proteins enriched in Group A (**Table 2**).

**Table 2:** Predicted type IV secretion system (T4SS) effector candidates and C-terminal E-Block–containing proteins in *C. jejuni*.

Protein ID	Protein annotation	Detection group	T4SS prediction (Secret4/S4TE)	E-Block motif	STRING functional context	Notes
TWO77289.1	Hypothetical protein E6O48_07200	Group A	Yes	Present	Uncharacterized	No conserved domains detected; strong acidic C-terminal tail
TWO77661.1	Peptidoglycan-associated lipoprotein Pal	Group A/Group B	Yes	Present	Tol–Pal system/cell envelope integrity	Outer membrane maintenance
TWO78479.1	Hypothetical protein E6O48_01875	Group A	Yes	Present	Uncharacterized	Predicted signal peptide; short acidic C-terminus
TWO77628.1	Cytolethal distending toxin subunit B (CdtB)	Group A	Yes	Present	CDT toxin complex (CdtA/B/C)	Dual T3SS/T4SS-like features
TWO75612.1	Phosphoglucosamine mutase PgmL	Group A	Yes	Present	Cell wall biosynthesis	Metabolic enzyme with non-canonical secretion signature
TWO78450.1	tRNA modification GTPase MnmE	Group A	Yes	Present	Translation/RNA modification	Non-classical secretion candidate
TWO78491.1	Invasion protein CiaB	Group A	Yes	Present	Host cell invasion module	Previously linked to secretion and invasion
TWO75222.1	Ankyrin repeat domain-containing protein	Group A	Yes	Present	Protein–protein interaction module	Eukaryotic-like domains

Immunoreactive proteins showing sequence features compatible with type IV secretion, integrating S4TE 2.0, SecReT4, and detection of C-terminal E-Block–like motifs.

While these features may be consistent with secretion-related signals described in other systems, it is important to note that *C. jejuni* lacks a well-characterized canonical T4SS, and no genomic or experimental evidence of such a secretion apparatus is currently available. Therefore, the identification of E-Block–like motifs and prediction-based classification should be interpreted with caution, as these observations are based solely on in silico analyses and do not demonstrate secretion or effector function.

### Quantitative differences in immune recognition intensity between Group A and Group B

Semi-quantitative estimation of protein abundance based on emPAI values showed a consistent immunodominance of stress response and housekeeping proteins in both groups (**Table 3**). Chaperonins such as GroEL and GroES displayed the highest emPAI values across all samples, in agreement with their well-established high abundance and strong immunogenicity. Comparative analysis of emPAI values revealed coherent differences between groups, with Group A sera exhibiting relatively higher representation of proteins associated with stress response, motility, and host interaction, including chemotaxis-related proteins, flagellar components (FlgE), and ribosome-associated chaperones such as Trigger factor and ClpB. In addition, proteins such as CdtB, CiaB, ankyrin repeat–containing proteins, and E-Block–bearing hypothetical proteins were detected or showed relatively higher representation in Group A samples. In contrast, proteins displaying similar emPAI values between groups were mainly associated with core metabolic processes and protein synthesis, reflecting background immune recognition likely driven by prior exposure. Taken together, these patterns support a more focused immune recognition profile during acute infection, particularly toward stress-adaptation and host interaction–related proteins, while remaining consistent with the semi-quantitative nature of the emPAI approach.

**Table 3:** Semi-quantitative emPAI profiling of immunoreactive proteins recognized by sera from Group A and Group B.

Protein ID	Protein name	Group B mean emPAI	Group A mean emPAI	A/B ratio	Functional category	Functional annotation/relevance
TWO77231.1	ClpB	0.42	0.59	1.40	Stress response	Chaperone, protein refolding (STRING)
TWO77289.1	Hypothetical protein	0.14	0.20	1.48	Secretion/Effector	Putative T4SS effector, no conserved domain
TWO77661.1	Pal	0.99	0.99	1.00	Cell envelope	Tol–Pal system, OM integrity, T4SS candidate
TWO78479.1	Hypothetical protein	0.76	1.05	1.38	Secretion/Effector	DNA-binding, T4SS candidate (E-Block)
TWO77628.1	CdtB	0.20	0.18	0.92	Virulence	Cytolethal distending toxin, T3SS substrate
TWO75612.1	PgmL	0.04	0.04	1.00	Metabolism	Cell envelope precursor synthesis
TWO78450.1	MnmE	0.00	0.09	∞	Translation	tRNA modification, stress adaptation
TWO78491.1	CiaB	0.00	0.03	∞	Virulence	Invasion protein, T3SS-associated
TWO78632.1	DnaK	0.81	0.73	0.91	Stress response	Heat-shock protein
TWO78203.1	GroEL	2.55	2.17	0.85	Stress response	Chaperonin, immunodominant antigen
TWO78204.1	GroES	2.90	2.90	1.00	Stress response	Co-chaperonin
TWO73462.1	Chemotaxis protein	1.47	1.99	1.35	Motility/VF	Chemotaxis, host colonization
TWO73463.1	FlaA	1.13	1.03	0.91	Motility/VF	Major flagellin
TWO75123.1	PorA	1.84	1.82	0.99	Outer membrane	Major porin, antigenic variability
TWO77259.1	Ribosomal protein S5	2.14	2.76	1.29	Protein synthesis	Ribosomal protein
TWO76322.1	HslJ	0.79	0.79	1.00	Stress response	Heat shock–related protein
TWO77230.1	Trigger factor	0.68	0.94	1.38	Protein folding	Ribosome-associated chaperone
TWO77109.1	Inorganic phosphatase	1.58	1.26	0.80	Metabolism	Phosphate metabolism
TWO77260.1	Ribosomal protein L18	1.66	2.16	1.30	Protein synthesis	Ribosomal protein
TWO77266.1	Ribosomal protein L14	0.89	1.26	1.42	Protein synthesis	Ribosomal protein
TWO77267.1	Ribosomal protein S17	1.78	2.25	1.26	Protein synthesis	Ribosomal protein
TWO77268.1	Ribosomal protein L29	1.51	1.51	1.00	Protein synthesis	Ribosomal protein
TWO77270.1	Ribosomal protein S3	1.99	1.81	0.91	Protein synthesis	Ribosomal protein
TWO77295.1	FlgE	0.08	0.11	1.38	Motility/VF	Flagellar hook protein
TWO77344.1	FliL	0.67	0.67	1.00	Motility/VF	Flagellar basal body
TWO77840.1	CheW	0.22	0.10	0.44	Motility	Chemotaxis coupling protein
TWO77742.1	KDO synthase	0.24	0.29	1.23	LOS/LPS synthesis	Core LPS biosynthesis
TWO73471.1	α-2,3-sialyltransferase	0.08	0.07	0.93	Virulence	LOS sialylation, molecular mimicry
TWO73476.1	SIS-domain protein	0.21	0.21	1.00	LOS synthesis	LPS biosynthesis
TWO73477.1	Dehydrogenase	0.14	0.13	0.93	Metabolism	Central metabolism
TWO74164.1	Zinc ribbon protein	0.12	0.12	1.00	Virulence	DNA-binding
TWO75222.1	Ankyrin repeat protein	0.14	0.13	0.94	Secretion/Effector	T4SS candidate, actin interaction

## Discussion

The present study provides an integrated immunoproteomic characterization of *C. jejuni* during acute campylobacteriosis (Group A) compared with asymptomatic individuals (Group B), combining immunocapture–based proteomics, semi-quantitative abundance estimation, and in silico functional and secretion system analyses. Our results reveal a largely shared antigenic background between both groups, accompanied by discrete but biologically meaningful differences associated with active infection.

The predominance of metabolic enzymes, ribosomal proteins, and stress-associated chaperones among immunoreactive targets in both groups is consistent with previous proteomic and immunoproteomic studies of *C. jejuni*, which have repeatedly shown that abundant housekeeping proteins dominate the humoral response regardless of clinical status (Cordwell et al., 2008; Scott and Cordwell, 2009; Nielsen et al., 2013). These proteins likely represent a conserved core antigenic repertoire arising from repeated exposure, bacterial turnover, and high intracellular abundance.

Despite this extensive overlap, the emPAI-based semi-quantitative analysis revealed infection-associated shifts in immune recognition intensity rather than a complete qualitative remodeling of the antigenic repertoire. Several proteins displayed higher relative abundance or preferential detection in Group A sera, particularly those associated with stress response, motility, secretion, and virulence. This pattern supports the concept that acute campylobacteriosis is characterized by selective enhancement of immune recognition driven by bacterial physiology during infection, rather than by the presence of unique virulence determinants alone (Young et al., 2007; Bolton, 2015).

A central finding of this work is the prominent immune recognition of the cytolethal distending toxin subunit CdtB in Group A sera. In addition, in silico analyses identified CdtB (TWO77628.1) as a candidate protein with features compatible with Type III secretion system (T3SS)-associated export. While *C. jejuni* is known to utilize the flagellar export apparatus for the secretion of certain virulence-associated proteins (Konkel et al., 2004; Neal-McKinney and Konkel, 2012), the classification of CdtB in this context is based on predictive tools and should be interpreted with caution, as no direct evidence of its secretion or translocation was assessed in the present study. Nevertheless, the established role of CdtB as part of the cytolethal distending toxin complex, which induces DNA damage and cell cycle arrest in host cells, supports its biological relevance during infection (Lara-Tejero and Galán, 2001).

STRING network analysis also highlighted the multidrug efflux system CmeABC, particularly the outer membrane component CmeC, as a major interaction hub recognized during acute disease. The CmeABC is classically associated with antimicrobial resistance, bile tolerance, and intestinal colonization, accumulating evidence indicates that efflux systems contribute directly to host adaptation and virulence (Lin et al., 2002; Luangtongkum et al., 2009). The consistent immune recognition and high emPAI values of CmeC and associated components in Group A sera suggest that these proteins are exposed or upregulated during infection, linking resistance mechanisms with virulence-associated antigenicity.

Flagellar and chemotaxis proteins, including FlgE, FlaA, CheW, and related components, were also prominently detected and displayed elevated emPAI values during acute infection. This observation aligns with the established role of motility and chemotaxis in epithelial invasion, colonization, and immune stimulation in *C. jejuni* (Barrero-Tobón and Hendrixson, 2014). The dual function of the flagellum as a motility organelle and secretion platform provides a mechanistic explanation for the strong immune targeting of these proteins during disease.

One of the most intriguing findings of this study is the identification of multiple immunoreactive proteins carrying sequence features compatible with those described for Type IV secretion system (T4SS) substrates in other bacterial pathogens. These proteins were identified using prediction tools such as Secret4 and S4TE and include Pal (TWO77661.1), CiaB (TWO78491.1), CdtB (TWO77628.1), ankyrin repeat–containing proteins (TWO75222.1), and several hypothetical proteins enriched in Group A. In particular, the presence of C-terminal acidic E-Block–like motifs, previously associated with T4SS substrates in organisms such as *L. pneumophila* and *C. burnetii* is noteworthy (Nagai et al., 2005; Burstein et al., 2009). However, it is important to emphasize that *C. jejuni* lacks a well-characterized canonical T4SS, and no genomic or experimental evidence currently supports the existence of such a secretion system in this organism. Therefore, these observations should be interpreted cautiously and considered as indicative of sequence features that may be functionally relevant, but that require experimental validation.

The detection of E-Block–containing proteins such as Pal, CiaB, ankyrin repeat–containing proteins, and additional hypothetical proteins enriched in Group A sera suggests that *C. jejuni* may exploit alternative secretion routes or multifunctional export systems to deliver host-interacting proteins. Pal, for instance, is a component of the Tol-Pal system involved in outer membrane stability and cell division, but has also been implicated in host interaction and immune stimulation. The presence of E-Block motifs and immune recognition during acute infection raises the possibility that these proteins participate in secretion or surface exposure events not yet fully characterized in *C. jejuni*.

Stress-associated chaperones such as GroEL, GroES, DnaK, and ClpB showed high emPAI values in both groups, but were particularly prominent during acute infection. Chaperones are among the most immunogenic bacterial proteins and are frequently upregulated under host-associated stress conditions, including oxidative stress, temperature shifts, and nutrient limitation (Henderson et al., 2006). Their strong immune recognition likely reflects bacterial adaptation to the hostile intestinal environment during disease.

In contrast, proteins uniquely or predominantly recognized in asymptomatic controls were mainly associated with basal metabolism and ribosomal function, consistent with immune memory generated by prior exposure or subclinical colonization. This supports a model in which disease outcome reflects dynamic host–pathogen interactions and differential protein expression or exposure, rather than the presence or absence of specific virulence genes.

To our knowledge, this is the first time that *in vivo*-induced antigens from *C. jejuni* an immunoproteomic approach combined immunocapture of *Campylobacter* proteins followed by nano–liquid chromatography–tandem mass spectrometry analysis. Previously, Hu et al. (2013) obtained a limited number of *in vivo*-induced antigens by applying an immunoproteomics method (2D electrophoresis coupled to western blotting and MALDI TOF MS) for identifying *in vivo*-induced *C. jejuni* antigens using pre-adsorbed human sera from infected patients. Our study has similar drawbacks due to limitations in the experimental design, for instance, it depends on which protein is actually immunogenic, false positives can arise because of immunological cross-reactivity, and also it ignores those genes that are expressed both *in vitro* and *in vivo*. However, those authors focused mainly in surface proteins and also recognized that their method requires the use of sera with very high titers, and that the majority of proteins cannot be detected on the PVDF membranes from 2-DE gels due to their low abundances. In contrast, ours is a cutting edge methodology more sensitive which allowed us to detect a higher number of immunogenic proteins independently of their abundance, some of them not previously associated with *Campylobacter* infection which warrants future studies to demonstrate their role in campylobacteriosis.

In summary, the integration of immunoproteomics, emPAI-based abundance estimation, STRING network analysis, and secretion system prediction reveals that acute campylobacteriosis is associated with enhanced immune recognition of stress-related, motility-associated, and secretion-linked proteins. The identification of flagellar T3SS substrates and T4SS-like effector candidates carrying E-Block motifs provides new insight into the complexity of protein export and host interaction strategies in *C. jejuni*, highlighting potential targets for future functional studies and diagnostic development.

## Data Availability

The datasets presented in this study can be found in online repositories. The names of the repository/repositories and accession number(s) can be found in the article/**Supplementary Material**.
