# Supplementary material for: Comparative immunoproteomics of Campylobacter jejuni in acute campylobacteriosis and asymptomatic individuals
Source: Front Cell Infect Microbiol. 2026 May 28;16:1800725. doi: 10.3389/fcimb.2026.1800725 (PMC13253429; doi:10.3389/fcimb.2026.1800725)
Supplement: Supplementary file 1 [file Table1.docx]

# SUPPLEMENTAL MATERIAL

# Supplementary Table S1: Functional Assignment of Groups A and B Proteins

| **Protein ID** | **Protein Name** | **Assigned Subsystem** | **Group** |
| --- | --- | --- | --- |
| TWO73462.1 | methyl-accepting chemotaxis protein, partial | Motility & Chemotaxis | A |
| TWO73463.1 | flagellin, partial | Motility & Chemotaxis | A |
| TWO73471.1 | alpha-2,3-sialyltransferase, partial | Metabolism | A |
| TWO73476.1 | SIS domain-containing protein | Other / Unknown | A |
| TWO73477.1 | dehydrogenase | Metabolism | A |
| TWO73518.1 | N-acetyl sugar amidotransferase | Metabolism | A |
| TWO73582.1 | pyridoxal phosphate-dependent aminotransferase | Metabolism | A |
| TWO73660.1 | PLP-dependent transferase | Metabolism | A |
| TWO73803.1 | methyl-accepting chemotaxis protein | Motility & Chemotaxis | A |
| TWO74228.1 | phosphatidylserine decarboxylase | Metabolism | A |
| TWO74309.1 | methyl-accepting chemotaxis protein, partial | Motility & Chemotaxis | A |
| TWO74341.1 | molybdopterin molybdotransferase MoeA | Metabolism | A |
| TWO74435.1 | gluconate 2-dehydrogenase subunit 3 family protein | Metabolism | A |
| TWO74445.1 | SPOR domain-containing protein | Other / Unknown | A |
| TWO74451.1 | Asp-tRNA(Asn)/Glu-tRNA(Gln) amidotransferase subunit GatC | Protein synthesis | A |
| TWO74461.1 | tryptophan--tRNA ligase | Protein synthesis | A |
| TWO74581.1 | cytochrome C | Other / Unknown | A |
| TWO74584.1 | methyl-accepting chemotaxis protein, partial | Motility & Chemotaxis | A |
| TWO74641.1 | aldehyde dehydrogenase | Metabolism | A |
| TWO74684.1 | 2-oxoglutarate ferredoxin oxidoreductase subunit beta | Other / Unknown | A |
| TWO74907.1 | multidrug efflux RND transporter outer membrane subunit CmeD | Cell envelope / Periplasm | A |
| TWO74943.1 | single-stranded DNA-binding protein | Other / Unknown | A |
| TWO75036.1 | DUF3373 family protein | Other / Unknown | A |
| TWO75123.1 | group 1 major outer membrane porin protein PorA | Cell envelope / Periplasm | A |
| TWO75222.1 | ankyrin repeat domain-containing protein | Other / Unknown | A |
| TWO75382.1 | periplasmic nitrate reductase subunit alpha | Other / Unknown | A |
| TWO75454.1 | flagellar hook protein FlgE | Motility & Chemotaxis | A |
| TWO75612.1 | phosphoglucosamine mutase PgmL | Other / Unknown | A |
| TWO75623.1 | adenylosuccinate lyase | Metabolism | A |
| TWO75775.1 | FkbM family methyltransferase | Metabolism | A |
| TWO75898.1 | 50S ribosomal protein L22 | Protein synthesis | A |
| TWO75950.1 | FTR1 family iron permease | Membrane transport & Secretion | A |
| TWO76034.1 | NADH-quinone oxidoreductase subunit NuoI | Other / Unknown | A |
| TWO76045.1 | methyl-accepting chemotaxis protein, partial | Motility & Chemotaxis | A |
| TWO76052.1 | 4-oxalocrotonate tautomerase family protein | Other / Unknown | A |
| TWO76074.1 | 3-methyl-2-oxobutanoate hydroxymethyltransferase | Metabolism | A |
| TWO76322.1 | META domain-containing protein | Other / Unknown | A |
| TWO76328.1 | phosphoribosylformylglycinamidine synthase subunit PurL | Metabolism | A |
| TWO76358.1 | porphobilinogen synthase | Metabolism | A |
| TWO76399.1 | type VI secretion system lipoprotein TssJ | Cell envelope / Periplasm | A |
| TWO76401.1 | type VI secretion system contractile sheath small subunit | Membrane transport & Secretion | A |
| TWO76402.1 | type VI secretion system contractile sheath large subunit | Membrane transport & Secretion | A |
| TWO76452.1 | 50S ribosomal protein L7/L12 | Protein synthesis | A |
| TWO76625.1 | acetate kinase | Metabolism | A |
| TWO76752.1 | major outer membrane protein | Cell envelope / Periplasm | A |
| TWO76840.1 | DUF3373 family protein | Other / Unknown | A |
| TWO76860.1 | D-glycero-beta-D-manno-heptose-7-phosphate kinase | Metabolism | A |
| TWO76993.1 | SDR family oxidoreductase | Other / Unknown | A |
| TWO77020.1 | SDR family oxidoreductase | Other / Unknown | A |
| TWO77045.1 | NADH-quinone oxidoreductase subunit G | Other / Unknown | A |
| TWO77060.1 | polysaccharide export protein | Other / Unknown | A |
| TWO77077.1 | superoxide dismutase | Other / Unknown | A |
| TWO77090.1 | 50S ribosomal protein L31 | Protein synthesis | A |
| TWO77095.1 | LL-diaminopimelate aminotransferase | Metabolism | A |
| TWO77098.1 | thioredoxin | Other / Unknown | A |
| TWO77099.1 | thioredoxin-disulfide reductase | Other / Unknown | A |
| TWO77104.1 | ketol-acid reductoisomerase | Cell envelope / Periplasm | A |
| TWO77109.1 | inorganic diphosphatase | Other / Unknown | A |
| TWO77110.1 | adenylate kinase | Metabolism | A |
| TWO77111.1 | aspartate--tRNA ligase | Protein synthesis | A |
| TWO77113.1 | DNA repair protein RecN | Other / Unknown | A |
| TWO77114.1 | PleD family two-component system response regulator | Cell envelope / Periplasm | A |
| TWO77117.1 | septal ring lytic transglycosylase RlpA family protein | Other / Unknown | A |
| TWO77186.1 | HDOD domain-containing protein | Other / Unknown | A |
| TWO77189.1 | 50S ribosomal protein L20 | Protein synthesis | A |
| TWO77194.1 | iron-sulfur cluster assembly scaffold protein NifU | Other / Unknown | A |
| TWO77195.1 | mechanosensitive ion channel family protein | Other / Unknown | A |
| TWO77202.1 | ribonucleotide-diphosphate reductase subunit beta | Other / Unknown | A |
| TWO77217.1 | translation initiation factor IF-3 | Other / Unknown | A |
| TWO77229.1 | GTP cyclohydrolase I FolE | Other / Unknown | A |
| TWO77230.1 | trigger factor | Other / Unknown | A |
| TWO77231.1 | ATP-dependent Clp protease proteolytic subunit | Other / Unknown | A |
| TWO77252.1 | citrate synthase | Metabolism | A |
| TWO77258.1 | 50S ribosomal protein L15 | Protein synthesis | A |
| TWO77259.1 | 30S ribosomal protein S5 | Protein synthesis | A |
| TWO77260.1 | 50S ribosomal protein L18 | Protein synthesis | A |
| TWO77261.1 | 50S ribosomal protein L6 | Protein synthesis | A |
| TWO77262.1 | 30S ribosomal protein S8 | Protein synthesis | A |
| TWO77264.1 | 50S ribosomal protein L5 | Protein synthesis | A |
| TWO77265.1 | 50S ribosomal protein L24 | Protein synthesis | A |
| TWO77266.1 | 50S ribosomal protein L14 | Protein synthesis | A |
| TWO77267.1 | 30S ribosomal protein S17 | Protein synthesis | A |
| TWO77268.1 | 50S ribosomal protein L29 | Protein synthesis | A |
| TWO77270.1 | 30S ribosomal protein S3 | Protein synthesis | A |
| TWO77272.1 | 30S ribosomal protein S19 | Protein synthesis | A |
| TWO77273.1 | 50S ribosomal protein L2 | Protein synthesis | A |
| TWO77274.1 | 50S ribosomal protein L23 | Protein synthesis | A |
| TWO77275.1 | 50S ribosomal protein L4 | Protein synthesis | A |
| TWO77276.1 | 50S ribosomal protein L3 | Protein synthesis | A |
| TWO77277.1 | 30S ribosomal protein S10 | Protein synthesis | A |
| TWO77279.1 | ribonuclease J | DNA/RNA metabolism | A |
| TWO77288.1 | 2-isopropylmalate synthase | Metabolism | A |
| TWO77292.1 | NAD(P)/FAD-dependent oxidoreductase | Other / Unknown | A |
| TWO77294.1 | O-acetylhomoserine aminocarboxypropyltransferase/cysteine synthase | Metabolism | A |
| TWO77295.1 | flagellar hook protein FlgE | Motility & Chemotaxis | A |
| TWO77298.1 | DNA polymerase III subunit beta | DNA/RNA metabolism | A |
| TWO77299.1 | DNA topoisomerase (ATP-hydrolyzing) subunit B | Metabolism | A |
| TWO77303.1 | glutamate synthase large subunit | Metabolism | A |
| TWO77304.1 | glutamate synthase subunit beta | Metabolism | A |
| TWO77307.1 | desulfoferrodoxin FeS4 iron-binding domain-containing protein | Other / Unknown | A |
| TWO77311.1 | 7-cyano-7-deazaguanine synthase QueC | Metabolism | A |
| TWO77312.1 | disulfide bond formation protein B | Other / Unknown | A |
| TWO77315.1 | cytochrome-c peroxidase | Metabolism | A |
| TWO77318.1 | adenylosuccinate lyase | Metabolism | A |
| TWO77319.1 | ribonucleoside-diphosphate reductase subunit alpha | Other / Unknown | A |
| TWO77321.1 | FAD-dependent thymidylate synthase | Metabolism | A |
| TWO77322.1 | CTP synthase | Metabolism | A |
| TWO77325.1 | radical SAM/SPASM domain-containing protein | Other / Unknown | A |
| TWO77332.1 | class I SAM-dependent methyltransferase | Metabolism | A |
| TWO77333.1 | methyltransferase domain-containing protein | Metabolism | A |
| TWO77344.1 | flagellar basal body-associated protein FliL | Motility & Chemotaxis | A |
| TWO77347.1 | type I glyceraldehyde-3-phosphate dehydrogenase | Metabolism | A |
| TWO77348.1 | phosphoglycerate kinase | Metabolism | A |
| TWO77350.1 | enoyl-ACP reductase FabI | Other / Unknown | A |
| TWO77357.1 | catalase | Other / Unknown | A |
| TWO77362.1 | DsbA family protein | Cell envelope / Periplasm | A |
| TWO77377.1 | class II fumarate hydratase | Other / Unknown | A |
| TWO77383.1 | ammonia-forming cytochrome c nitrite reductase subunit c552 | Other / Unknown | A |
| TWO77385.1 | siderophore ABC transporter substrate-binding protein | Membrane transport & Secretion | A |
| TWO77394.1 | 1-deoxy-D-xylulose-5-phosphate reductoisomerase | Metabolism | A |
| TWO77400.1 | glutamine--fructose-6-phosphate transaminase (isomerizing) | Other / Unknown | A |
| TWO77413.1 | 30S ribosomal protein S13 | Protein synthesis | A |
| TWO77414.1 | 30S ribosomal protein S11 | Protein synthesis | A |
| TWO77415.1 | 30S ribosomal protein S4 | Protein synthesis | A |
| TWO77416.1 | DNA-directed RNA polymerase subunit alpha | DNA/RNA metabolism | A |
| TWO77417.1 | 50S ribosomal protein L17 | Protein synthesis | A |
| TWO77418.1 | ATP phosphoribosyltransferase | Metabolism | A |
| TWO77419.1 | histidinol dehydrogenase | Metabolism | A |
| TWO77420.1 | bifunctional histidinol-phosphatase/imidazoleglycerol-phosphate dehydratase HisB | Other / Unknown | A |
| TWO77427.1 | MRP family ATP-binding protein | Other / Unknown | A |
| TWO77432.1 | 30S ribosomal protein S20 | Protein synthesis | A |
| TWO77445.1 | amino acid transporter | Membrane transport & Secretion | A |
| TWO77461.1 | UDP-N-acetylmuramoyl-L-alanyl-D-glutamate--2,6-diaminopimelate ligase | DNA/RNA metabolism | A |
| TWO77463.1 | PDZ domain-containing protein | Other / Unknown | A |
| TWO77489.1 | phosphopyruvate hydratase | Other / Unknown | A |
| TWO77490.1 | recombinase RecA | Other / Unknown | A |
| TWO77516.1 | pyruvate:ferredoxin (flavodoxin) oxidoreductase | Other / Unknown | A |
| TWO77518.1 | fibronectin-binding outer membrane protein CadF | Cell envelope / Periplasm | A |
| TWO77519.1 | 30S ribosomal protein S9 | Protein synthesis | A |
| TWO77520.1 | 50S ribosomal protein L13 | Protein synthesis | A |
| TWO77527.1 | c-type cytochrome | Other / Unknown | A |
| TWO77529.1 | cytochrome-c oxidase, cbb3-type subunit II | Metabolism | A |
| TWO77530.1 | cytochrome-c oxidase, cbb3-type subunit I | Metabolism | A |
| TWO77542.1 | aldehyde dehydrogenase family protein | Metabolism | A |
| TWO77545.1 | methyl-accepting chemotaxis protein | Motility & Chemotaxis | A |
| TWO77547.1 | formate dehydrogenase accessory sulfurtransferase FdhD | Metabolism | A |
| TWO77549.1 | 4Fe-4S dicluster domain-containing protein | Other / Unknown | A |
| TWO77550.1 | formate dehydrogenase | Metabolism | A |
| TWO77554.1 | multicopper oxidase CueO | Metabolism | A |
| TWO77560.1 | phosphoribosylformylglycinamidine cyclo-ligase | DNA/RNA metabolism | A |
| TWO77565.1 | DNA starvation/stationary phase protection protein | Other / Unknown | A |
| TWO77568.1 | acetate--CoA ligase | DNA/RNA metabolism | A |
| TWO77572.1 | LamB/YcsF family protein | Other / Unknown | A |
| TWO77591.1 | SIMPL domain-containing protein | Other / Unknown | A |
| TWO77594.1 | c-type cytochrome | Other / Unknown | A |
| TWO77596.1 | translational GTPase TypA | Other / Unknown | A |
| TWO77607.1 | tRNA 2-thiouridine(34) synthase MnmA | Protein synthesis | A |
| TWO77617.1 | flagellar biosynthesis protein FlhF | Other / Unknown | A |
| TWO77622.1 | Cj0069 family protein | Other / Unknown | A |
| TWO77623.1 | lactate utilization protein C | Other / Unknown | A |
| TWO77624.1 | iron-sulfur cluster-binding protein | Other / Unknown | A |
| TWO77625.1 | (Fe-S)-binding protein | Other / Unknown | A |
| TWO77628.1 | cytolethal distending toxin nuclease subunit Cj-CdtB | DNA/RNA metabolism | A |
| TWO77631.1 | cytochrome ubiquinol oxidase subunit I | Metabolism | A |
| TWO77635.1 | aspartate ammonia-lyase | Metabolism | A |
| TWO77636.1 | anaerobic C4-dicarboxylate transporter | Membrane transport & Secretion | A |
| TWO77639.1 | penicillin-binding protein activator LpoB | Other / Unknown | A |
| TWO77642.1 | 50S ribosomal protein L21 | Protein synthesis | A |
| TWO77643.1 | 50S ribosomal protein L27 | Protein synthesis | A |
| TWO77649.1 | ParB/RepB/Spo0J family partition protein | Other / Unknown | A |
| TWO77651.1 | F0F1 ATP synthase subunit B | Metabolism | A |
| TWO77652.1 | F0F1 ATP synthase subunit delta | Metabolism | A |
| TWO77653.1 | F0F1 ATP synthase subunit alpha | Metabolism | A |
| TWO77654.1 | F0F1 ATP synthase subunit gamma | Metabolism | A |
| TWO77655.1 | F0F1 ATP synthase subunit beta | Metabolism | A |
| TWO77660.1 | Tol-Pal system protein TolB | Cell envelope / Periplasm | A |
| TWO77661.1 | peptidoglycan-associated lipoprotein Pal | Cell envelope / Periplasm | A |
| TWO77662.1 | tetratricopeptide repeat protein | Other / Unknown | A |
| TWO77675.1 | acetyl-CoA carboxylase carboxyltransferase subunit beta | Metabolism | A |
| TWO77677.1 | outer membrane protein assembly factor BamA | Cell envelope / Periplasm | A |
| TWO77679.1 | M23 family metallopeptidase | Other / Unknown | A |
| TWO77684.1 | translation initiation factor IF-2 | Other / Unknown | A |
| TWO77687.1 | McrB family protein | Other / Unknown | A |
| TWO77691.1 | ABC transporter substrate-binding protein | Membrane transport & Secretion | A |
| TWO77692.1 | methyl-accepting chemotaxis protein, partial | Motility & Chemotaxis | A |
| TWO77700.1 | ABC-F family ATP-binding cassette domain-containing protein | Other / Unknown | A |
| TWO77706.1 | polyisoprenoid-binding protein | Other / Unknown | A |
| TWO77711.1 | GMC family oxidoreductase | Other / Unknown | A |
| TWO77716.1 | fumarate reductase iron-sulfur subunit | Other / Unknown | A |
| TWO77717.1 | fumarate reductase flavoprotein subunit | Other / Unknown | A |
| TWO77720.1 | lipoprotein | Cell envelope / Periplasm | A |
| TWO77724.1 | serine hydroxymethyltransferase | Metabolism | A |
| TWO77725.1 | lysine--tRNA ligase | Protein synthesis | A |
| TWO77730.1 | lipoprotein | Cell envelope / Periplasm | A |
| TWO77733.1 | FAD-dependent oxidoreductase | Other / Unknown | A |
| TWO77734.1 | pyruvate kinase | Metabolism | A |
| TWO77740.1 | ribosome biogenesis GTPase Der | Other / Unknown | A |
| TWO77742.1 | 3-deoxy-8-phosphooctulonate synthase | Metabolism | A |
| TWO77743.1 | 6,7-dimethyl-8-ribityllumazine synthase | Metabolism | A |
| TWO77753.1 | D-2-hydroxyacid dehydrogenase | Metabolism | A |
| TWO77756.1 | 30S ribosomal protein S21 | Protein synthesis | A |
| TWO77759.1 | multidrug efflux RND transporter periplasmic adaptor subunit CmeA | Membrane transport & Secretion | A |
| TWO77760.1 | multidrug efflux RND transporter permease subunit CmeB | Membrane transport & Secretion | A |
| TWO77761.1 | multidrug efflux transporter outer membrane subunit CmeC | Cell envelope / Periplasm | A |
| TWO77766.1 | phosphoglucosamine mutase | Other / Unknown | A |
| TWO77767.1 | cytochrome-c peroxidase | Metabolism | A |
| TWO77770.1 | homeostatic response regulator transcription factor HsrA | DNA/RNA metabolism | A |
| TWO77772.1 | Ppx/GppA family phosphatase | Other / Unknown | A |
| TWO77788.1 | flagellar motor protein MotA | Motility & Chemotaxis | A |
| TWO77791.1 | peroxiredoxin | Other / Unknown | A |
| TWO77793.1 | nucleoside-diphosphate kinase | Metabolism | A |
| TWO77797.1 | ketoacyl-ACP synthase III | Metabolism | A |
| TWO77806.1 | flagellar motor switch protein FliG | Motility & Chemotaxis | A |
| TWO77807.1 | flagellar basal body M-ring protein FliF | Motility & Chemotaxis | A |
| TWO77814.1 | 50S ribosomal protein L25 | Protein synthesis | A |
| TWO77829.1 | pantoate--beta-alanine ligase | DNA/RNA metabolism | A |
| TWO77833.1 | 5'/3'-nucleotidase SurE | Other / Unknown | A |
| TWO77834.1 | AcfC family glycoprotein adhesin PEB3 | Virulence / Defense | A |
| TWO77839.1 | hybrid sensor histidine kinase/response regulator | Metabolism | A |
| TWO77840.1 | purine-binding chemotaxis protein CheW | Motility & Chemotaxis | A |
| TWO77844.1 | carbamoyl-phosphate synthase large subunit | Metabolism | A |
| TWO77846.1 | rod shape-determining protein | Other / Unknown | A |
| TWO77847.1 | ATP-dependent Clp protease ATP-binding subunit ClpX | Other / Unknown | A |
| TWO77853.1 | branched-chain-amino-acid transaminase | Other / Unknown | A |
| TWO77854.1 | prohibitin family protein | Other / Unknown | A |
| TWO77858.1 | molybdopterin guanine dinucleotide-containing S/N-oxide reductase | Other / Unknown | A |
| TWO77860.1 | methyl-accepting chemotaxis protein, partial | Motility & Chemotaxis | A |
| TWO77865.1 | hydrogenase accessory protein HypB | Other / Unknown | A |
| TWO77874.1 | non-heme ferritin | Other / Unknown | A |
| TWO77883.1 | protein-disulfide reductase DsbD | Cell envelope / Periplasm | A |
| TWO77889.1 | class II fructose-bisphosphate aldolase | Other / Unknown | A |
| TWO77890.1 | peptidylprolyl isomerase PEB4 | Virulence / Defense | A |
| TWO77903.1 | aspartate kinase | Metabolism | A |
| TWO77910.1 | acetolactate synthase small subunit | Cell envelope / Periplasm | A |
| TWO77913.1 | 3,4-dihydroxy-2-butanone-4-phosphate synthase | Metabolism | A |
| TWO77926.1 | cbb3-type cytochrome oxidase assembly protein CcoS | Metabolism | A |
| TWO77942.1 | proline--tRNA ligase | Protein synthesis | A |
| TWO77943.1 | glutamyl-tRNA reductase | Protein synthesis | A |
| TWO77944.1 | hexaprenyl-diphosphate synthase | Metabolism | A |
| TWO77947.1 | 2-oxoglutarate:acceptor oxidoreductase | Other / Unknown | A |
| TWO77949.1 | 2-oxoglutarate synthase subunit alpha | Metabolism | A |
| TWO77951.1 | succinate--CoA ligase subunit alpha | DNA/RNA metabolism | A |
| TWO77952.1 | ADP-forming succinate--CoA ligase subunit beta | DNA/RNA metabolism | A |
| TWO77954.1 | NADP-dependent isocitrate dehydrogenase | Metabolism | A |
| TWO77963.1 | molecular chaperone HtpG | Stress response | A |
| TWO77967.1 | phosphoribosylformylglycinamidine synthase I | Metabolism | A |
| TWO77968.1 | phosphoribosylformylglycinamidine synthase, purS protein | Metabolism | A |
| TWO77969.1 | phosphoribosylaminoimidazolesuccinocarboxamide synthase | Metabolism | A |
| TWO77970.1 | S41 family peptidase | Other / Unknown | A |
| TWO77972.1 | AAA family ATPase | Other / Unknown | A |
| TWO77975.1 | alanine--tRNA ligase | Protein synthesis | A |
| TWO77987.1 | elongation factor G | Protein synthesis | A |
| TWO77988.1 | 30S ribosomal protein S7 | Protein synthesis | A |
| TWO77989.1 | 30S ribosomal protein S12 | Protein synthesis | A |
| TWO77999.1 | DNA-directed RNA polymerase subunit beta' | DNA/RNA metabolism | A |
| TWO78000.1 | DNA-directed RNA polymerase subunit beta | DNA/RNA metabolism | A |
| TWO78002.1 | 50S ribosomal protein L10 | Protein synthesis | A |
| TWO78003.1 | 50S ribosomal protein L1 | Protein synthesis | A |
| TWO78004.1 | 50S ribosomal protein L11 | Protein synthesis | A |
| TWO78008.1 | elongation factor Tu | Protein synthesis | A |
| TWO78018.1 | transcription termination/antitermination protein NusA | DNA/RNA metabolism | A |
| TWO78025.1 | phosphomethylpyrimidine synthase ThiC | Metabolism | A |
| TWO78028.1 | 50S ribosomal protein L28 | Protein synthesis | A |
| TWO78030.1 | chemotaxis protein | Motility & Chemotaxis | A |
| TWO78033.1 | acetyl-CoA carboxylase carboxyl transferase subunit alpha | Metabolism | A |
| TWO78034.1 | beta-ketoacyl-ACP synthase II | Metabolism | A |
| TWO78035.1 | acyl carrier protein | Other / Unknown | A |
| TWO78036.1 | thiaminase II | Other / Unknown | A |
| TWO78041.1 | 3-oxoacyl-ACP reductase FabG | Other / Unknown | A |
| TWO78059.1 | molybdopterin adenylyltransferase | Metabolism | A |
| TWO78069.1 | hydroxyisourate hydrolase | Other / Unknown | A |
| TWO78070.1 | 50S ribosomal protein L19 | Protein synthesis | A |
| TWO78074.1 | 30S ribosomal protein S16 | Protein synthesis | A |
| TWO78075.1 | signal recognition particle protein | Other / Unknown | A |
| TWO78080.1 | glycine--tRNA ligase subunit alpha | Protein synthesis | A |
| TWO78085.1 | type I glutamate--ammonia ligase | DNA/RNA metabolism | A |
| TWO78089.1 | cell division protein FtsA | Other / Unknown | A |
| TWO78090.1 | peptidylprolyl isomerase | Metabolism | A |
| TWO78096.1 | phosphate acetyltransferase | Metabolism | A |
| TWO78098.1 | flavodoxin-dependent (E)-4-hydroxy-3-methylbut-2-enyl-diphosphate synthase | Metabolism | A |
| TWO78106.1 | anaerobic C4-dicarboxylate transporter | Membrane transport & Secretion | A |
| TWO78111.1 | argininosuccinate synthase | Metabolism | A |
| TWO78112.1 | 50S ribosomal protein L9 | Protein synthesis | A |
| TWO78114.1 | ATP-dependent protease ATPase subunit HslU | Other / Unknown | A |
| TWO78133.1 | UDP-N-acetylglucosamine 4,6-dehydratase (inverting) | Other / Unknown | A |
| TWO78139.1 | malate dehydrogenase | Metabolism | A |
| TWO78146.1 | fibronectin type III domain-containing protein | Other / Unknown | A |
| TWO78151.1 | UMP kinase | Metabolism | A |
| TWO78152.1 | DNA-directed RNA polymerase subunit omega | DNA/RNA metabolism | A |
| TWO78154.1 | tyrosine--tRNA ligase | Protein synthesis | A |
| TWO78155.1 | nitronate monooxygenase | Other / Unknown | A |
| TWO78158.1 | Ni/Fe hydrogenase | Other / Unknown | A |
| TWO78159.1 | nickel-dependent hydrogenase large subunit | Other / Unknown | A |
| TWO78164.1 | response regulator transcription factor | DNA/RNA metabolism | A |
| TWO78166.1 | major outer membrane protein | Cell envelope / Periplasm | A |
| TWO78171.1 | polyribonucleotide nucleotidyltransferase | Metabolism | A |
| TWO78174.1 | phosphoribosylamine--glycine ligase | DNA/RNA metabolism | A |
| TWO78176.1 | glutamine-hydrolyzing GMP synthase | Metabolism | A |
| TWO78196.1 | DegQ family serine endoprotease | Other / Unknown | A |
| TWO78197.1 | response regulator transcription factor | DNA/RNA metabolism | A |
| TWO78203.1 | chaperonin GroEL | Stress response | A |
| TWO78204.1 | co-chaperone GroES | Stress response | A |
| TWO78209.1 | M23 family metallopeptidase | Other / Unknown | A |
| TWO78218.1 | signal recognition particle-docking protein FtsY | Other / Unknown | A |
| TWO78219.1 | DNA repair protein RadA | Other / Unknown | A |
| TWO78226.1 | S-ribosylhomocysteine lyase | Metabolism | A |
| TWO78227.1 | Asp-tRNA(Asn)/Glu-tRNA(Gln) amidotransferase subunit GatB | Protein synthesis | A |
| TWO78240.1 | c-type cytochrome | Other / Unknown | A |
| TWO78242.1 | 30S ribosomal protein S2 | Protein synthesis | A |
| TWO78243.1 | elongation factor Ts | Protein synthesis | A |
| TWO78268.1 | transcription termination factor Rho | DNA/RNA metabolism | A |
| TWO78271.1 | cytochrome c | Other / Unknown | A |
| TWO78288.1 | UDP-glucose 4-epimerase GalE | Other / Unknown | A |
| TWO78301.1 | response regulator | Other / Unknown | A |
| TWO78303.1 | ATP-dependent zinc metalloprotease FtsH | Other / Unknown | A |
| TWO78308.1 | PAS domain-containing protein | Other / Unknown | A |
| TWO78310.1 | AAA family ATPase | Other / Unknown | A |
| TWO78312.1 | TlpA family protein disulfide reductase | Other / Unknown | A |
| TWO78322.1 | methionine adenosyltransferase | Metabolism | A |
| TWO78324.1 | preprotein translocase subunit YajC | Membrane transport & Secretion | A |
| TWO78325.1 | protein translocase subunit SecD | Membrane transport & Secretion | A |
| TWO78327.1 | leucine--tRNA ligase | Protein synthesis | A |
| TWO78331.1 | M23 family metallopeptidase | Other / Unknown | A |
| TWO78332.1 | polymer-forming cytoskeletal family protein | Other / Unknown | A |
| TWO78345.1 | endopeptidase La | Other / Unknown | A |
| TWO78352.1 | NAD(P)H-dependent oxidoreductase | Other / Unknown | A |
| TWO78356.1 | isoleucine--tRNA ligase | Protein synthesis | A |
| TWO78358.1 | Asp-tRNA(Asn)/Glu-tRNA(Gln) amidotransferase subunit GatA | Protein synthesis | A |
| TWO78359.1 | IMP dehydrogenase | Metabolism | A |
| TWO78368.1 | succinyl-diaminopimelate desuccinylase | Other / Unknown | A |
| TWO78371.1 | thiazole synthase | Metabolism | A |
| TWO78376.1 | undecaprenyldiphospho-muramoylpentapeptide beta-N-acetylglucosaminyltransferase | Metabolism | A |
| TWO78378.1 | acetyl-CoA carboxylase subunit A | Metabolism | A |
| TWO78382.1 | multidrug efflux RND transporter permease subunit CmeF | Membrane transport & Secretion | A |
| TWO78383.1 | multidrug efflux RND transporter periplasmic adaptor subunit CmeE | Membrane transport & Secretion | A |
| TWO78385.1 | elongation factor 4 | Protein synthesis | A |
| TWO78388.1 | DNA topoisomerase (ATP-hydrolyzing) subunit A | Metabolism | A |
| TWO78389.1 | lipoprotein | Cell envelope / Periplasm | A |
| TWO78391.1 | sigma-54-dependent Fis family transcriptional regulator | DNA/RNA metabolism | A |
| TWO78392.1 | aspartate-semialdehyde dehydrogenase | Metabolism | A |
| TWO78396.1 | ABC transporter substrate-binding protein | Membrane transport & Secretion | A |
| TWO78402.1 | cytochrome C biogenesis protein | Other / Unknown | A |
| TWO78407.1 | mechanosensitive ion channel | Other / Unknown | A |
| TWO78412.1 | RNA polymerase sigma factor RpoD | DNA/RNA metabolism | A |
| TWO78418.1 | porphobilinogen synthase | Metabolism | A |
| TWO78430.1 | transporter substrate-binding domain-containing protein | Membrane transport & Secretion | A |
| TWO78450.1 | tRNA uridine-5-carboxymethylaminomethyl(34) synthesis GTPase MnmE | Protein synthesis | A |
| TWO78463.1 | preprotein translocase subunit SecA | Membrane transport & Secretion | A |
| TWO78472.1 | biotin attachment protein | Other / Unknown | A |
| TWO78473.1 | phosphoenolpyruvate carboxykinase (ATP) | Metabolism | A |
| TWO78476.1 | leucyl aminopeptidase | Other / Unknown | A |
| TWO78483.1 | amino acid ABC transporter ATP-binding protein | Membrane transport & Secretion | A |
| TWO78484.1 | bifunctional adhesin/ABC transporter aspartate/glutamate-binding protein PEB1a | Membrane transport & Secretion | A |
| TWO78491.1 | invasion protein CiaB | Virulence / Defense | A |
| TWO78492.1 | DNA-binding protein HU | Other / Unknown | A |
| TWO78501.1 | sodium:alanine symporter family protein | Other / Unknown | A |
| TWO78507.1 | phenylalanine--tRNA ligase subunit alpha | Protein synthesis | A |
| TWO78508.1 | phenylalanine--tRNA ligase subunit beta | Protein synthesis | A |
| TWO78511.1 | 30S ribosomal protein S1 | Protein synthesis | A |
| TWO78513.1 | phosphoglycerate dehydrogenase | Metabolism | A |
| TWO78535.1 | UDP-N-acetylglucosamine 1-carboxyvinyltransferase | Metabolism | A |
| TWO78540.1 | glutamate-1-semialdehyde-2,1-aminomutase | Other / Unknown | A |
| TWO78558.1 | bifunctional aconitate hydratase 2/2-methylisocitrate dehydratase | Other / Unknown | A |
| TWO78560.1 | SDR family NAD(P)-dependent oxidoreductase | Other / Unknown | A |
| TWO78561.1 | Na+/H+ antiporter NhaC family protein | Other / Unknown | A |
| TWO78572.1 | bifunctional UDP-N-acetylglucosamine diphosphorylase/glucosamine-1-phosphate N-acetyltransferase GlmU | Metabolism | A |
| TWO78579.1 | threonine synthase | Metabolism | A |
| TWO78584.1 | enoyl-ACP reductase | Other / Unknown | A |
| TWO78585.1 | 4-hydroxy-tetrahydrodipicolinate synthase | Metabolism | A |
| TWO78603.1 | 2-oxoglutarate:acceptor oxidoreductase | Other / Unknown | A |
| TWO78608.1 | nitrate reductase cytochrome c-type subunit | Other / Unknown | A |
| TWO78612.1 | thiol peroxidase | Metabolism | A |
| TWO78619.1 | MetQ/NlpA family ABC transporter substrate-binding protein | Membrane transport & Secretion | A |
| TWO78620.1 | MetQ/NlpA family ABC transporter substrate-binding protein | Membrane transport & Secretion | A |
| TWO78621.1 | MetQ/NlpA family ABC transporter substrate-binding protein | Membrane transport & Secretion | A |
| TWO78626.1 | histidine--tRNA ligase | Protein synthesis | A |
| TWO78632.1 | molecular chaperone DnaK | Stress response | A |
| TWO75593.1 | class I SAM-dependent methyltransferase | Metabolism | A |
| TWO75635.1 | 4Fe-4S dicluster domain-containing protein | Other / Unknown | A |
| TWO76268.1 | 4-hydroxy-3-methylbut-2-enyl diphosphate reductase | Other / Unknown | A |
| TWO76321.1 | agamatine deiminase | Other / Unknown | A |
| TWO77182.1 | cyclic pyranopterin monophosphate synthase MoaC | Metabolism | A |
| TWO77254.1 | biotin synthase | Metabolism | A |
| TWO77306.1 | ComEA family DNA-binding protein | Other / Unknown | A |
| TWO77308.1 | dihydroxy-acid dehydratase | Other / Unknown | A |
| TWO77434.1 | HugZ family heme oxygenase | Other / Unknown | A |
| TWO77454.1 | chorismate synthase | Metabolism | A |
| TWO77531.1 | response regulator transcription factor | DNA/RNA metabolism | A |
| TWO77538.1 | adenylosuccinate synthase | Metabolism | A |
| TWO77571.1 | tungsten ABC transporter substrate-binding protein | Membrane transport & Secretion | A |
| TWO77616.1 | MinD/ParA family protein | Other / Unknown | A |
| TWO77648.1 | ParA family protein | Other / Unknown | A |
| TWO77737.1 | serine--tRNA ligase | Protein synthesis | A |
| TWO77863.1 | hydrogenase formation protein HypD | Other / Unknown | A |
| TWO77929.1 | amidohydrolase | Other / Unknown | A |
| TWO77953.1 | malate dehydrogenase | Metabolism | A |
| TWO78009.1 | amino acid ABC transporter ATP-binding protein | Membrane transport & Secretion | A |
| TWO78015.1 | insulinase family protein | Other / Unknown | A |
| TWO78047.1 | filamentous hemagglutinin N-terminal domain-containing protein | Other / Unknown | A |
| TWO78068.1 | 3-deoxy-7-phosphoheptulonate synthase class II | Metabolism | A |
| TWO78136.1 | acetyl-CoA carboxylase biotin carboxylase subunit | Metabolism | A |
| TWO78148.1 | ABC transporter ATP-binding protein | Membrane transport & Secretion | A |
| TWO78149.1 | ABC transporter permease | Membrane transport & Secretion | A |
| TWO78229.1 | metal-dependent hydrolase | Other / Unknown | A |
| TWO78409.1 | ATP-dependent metallopeptidase FtsH/Yme1/Tma family protein | Other / Unknown | A |
| TWO78422.1 | (Fe-S)-binding protein | Other / Unknown | A |
| TWO78454.1 | methyl-accepting chemotaxis protein | Motility & Chemotaxis | A |
| TWO78485.1 | amino acid ABC transporter permease | Membrane transport & Secretion | A |
| TWO78490.1 | acyl-CoA thioesterase | Other / Unknown | A |
| TWO78555.1 | methionine--tRNA ligase | Protein synthesis | A |
| TWO78569.1 | isoprenyl transferase | Metabolism | A |
| TWO78586.1 | insulinase family protein | Other / Unknown | A |
| TWO78625.1 | dTMP kinase | Metabolism | A |
| TWO75189.1 | flagellin, partial | Motility & Chemotaxis | B |
| TWO77255.1 | type I DNA topoisomerase | Metabolism | B |
| TWO77658.1 | ExbD/TolR family protein | Cell envelope / Periplasm | B |
| TWO78050.1 | basic amino acid ABC transporter substrate-binding protein | Membrane transport & Secretion | B |
| TWO73481.1 | glycosyltransferase | Metabolism | B |
| TWO73619.1 | phosphatidate cytidylyltransferase | Metabolism | B |
| TWO74143.1 | DUF2972 domain-containing protein | Other / Unknown | B |
| TWO74318.1 | NAD(P)-dependent alcohol dehydrogenase | Metabolism | B |
| TWO74942.1 | 30S ribosomal protein S6 | Protein synthesis | B |
| TWO75010.1 | UDP-N-acetylglucosamine 2-epimerase (hydrolyzing) | Other / Unknown | B |
| TWO75011.1 | acylneuraminate cytidylyltransferase family protein | Metabolism | B |
| TWO75225.1 | ankyrin repeat domain-containing protein | Other / Unknown | B |
| TWO75366.1 | serine O-acetyltransferase | Metabolism | B |
| TWO75722.1 | DUF1425 domain-containing protein | Other / Unknown | B |
| TWO76003.1 | HrgA protein | Other / Unknown | B |
| TWO76395.1 | type VI secretion system membrane subunit TssM | Membrane transport & Secretion | B |
| TWO76507.1 | flagellar filament capping protein FliD | Motility & Chemotaxis | B |
| TWO76579.1 | autotransporter | Membrane transport & Secretion | B |
| TWO76968.1 | flagellin, partial | Motility & Chemotaxis | B |
| TWO76996.1 | N-acetyl sugar amidotransferase | Metabolism | B |
| TWO77000.1 | UDP-2,4-diacetamido-2,4,6-trideoxy-beta-L-altropyranose hydrolase | Other / Unknown | B |
| TWO77033.1 | N,N'-diacetyllegionaminate synthase | Metabolism | B |
| TWO77061.1 | capsule biosynthesis protein | Other / Unknown | B |
| TWO77193.1 | cysteine desulfurase, NifS family | Other / Unknown | B |
| TWO77226.1 | 4-hydroxy-tetrahydrodipicolinate reductase | Other / Unknown | B |
| TWO77235.1 | bifunctional ADP-dependent NAD(P)H-hydrate dehydratase/NAD(P)H-hydrate epimerase | Other / Unknown | B |
| TWO77240.1 | HlyC/CorC family transporter | Membrane transport & Secretion | B |
| TWO77310.1 | HD family hydrolase | Other / Unknown | B |
| TWO77339.1 | capsule biosynthesis protein | Other / Unknown | B |
| TWO77364.1 | L-seryl-tRNA(Sec) selenium transferase | Protein synthesis | B |
| TWO77376.1 | autotransporter domain-containing protein | Membrane transport & Secretion | B |
| TWO77382.1 | cytochrome c nitrite reductase small subunit | Other / Unknown | B |
| TWO77403.1 | ABC transporter permease | Membrane transport & Secretion | B |
| TWO77412.1 | 50S ribosomal protein L36 | Protein synthesis | B |
| TWO77421.1 | imidazole glycerol phosphate synthase subunit HisH | Metabolism | B |
| TWO77444.1 | L-serine ammonia-lyase | Metabolism | B |
| TWO77453.1 | ATP-binding protein | Other / Unknown | B |
| TWO77471.1 | type I methionyl aminopeptidase | Other / Unknown | B |
| TWO77593.1 | DUF2130 domain-containing protein | Other / Unknown | B |
| TWO77612.1 | flagellar motor switch protein FliY | Motility & Chemotaxis | B |
| TWO77656.1 | F0F1 ATP synthase subunit epsilon | Metabolism | B |
| TWO77665.1 | 5'-methylthioadenosine/adenosylhomocysteine nucleosidase | Other / Unknown | B |
| TWO77718.1 | fumarate reductase cytochrome b subunit | Other / Unknown | B |
| TWO77736.1 | tetratricopeptide repeat protein | Other / Unknown | B |
| TWO77752.1 | YajQ family cyclic di-GMP-binding protein | Other / Unknown | B |
| TWO77768.1 | glycerol-3-phosphate 1-O-acyltransferase PlsY | Metabolism | B |
| TWO77776.1 | tryptophan synthase subunit alpha | Metabolism | B |
| TWO77783.1 | excinuclease ABC subunit UvrA | DNA/RNA metabolism | B |
| TWO77805.1 | flagellar assembly protein FliH | Motility & Chemotaxis | B |
| TWO77838.1 | chemotaxis protein CheV | Motility & Chemotaxis | B |
| TWO77841.1 | phosphoserine phosphatase SerB | Other / Unknown | B |
| TWO77851.1 | thioredoxin-dependent thiol peroxidase | Metabolism | B |
| TWO77880.1 | efflux RND transporter periplasmic adaptor subunit | Membrane transport & Secretion | B |
| TWO77881.1 | amidohydrolase | Other / Unknown | B |
| TWO77955.1 | DUF3971 domain-containing protein | Other / Unknown | B |
| TWO77973.1 | penicillin-binding protein 1A | Other / Unknown | B |
| TWO78005.1 | transcription termination/antitermination protein NusG | DNA/RNA metabolism | B |
| TWO78058.1 | magnesium/cobalt transporter CorA | Membrane transport & Secretion | B |
| TWO78065.1 | YggS family pyridoxal phosphate-dependent enzyme | Other / Unknown | B |
| TWO78076.1 | RluA family pseudouridine synthase | Metabolism | B |
| TWO78103.1 | excinuclease ABC subunit UvrB | DNA/RNA metabolism | B |
| TWO78134.1 | dCTP deaminase | Other / Unknown | B |
| TWO78138.1 | glutamate--tRNA ligase | Protein synthesis | B |
| TWO78142.1 | TrkA family potassium uptake protein | Other / Unknown | B |
| TWO78172.1 | LPS-assembly protein LptD | Other / Unknown | B |
| TWO78200.1 | bacteriohemerythrin | Other / Unknown | B |
| TWO78211.1 | FAD-binding protein | Other / Unknown | B |
| TWO78215.1 | ribonuclease Y | DNA/RNA metabolism | B |
| TWO78246.1 | highly acidic protein | Other / Unknown | B |
| TWO78252.1 | YebC/PmpR family DNA-binding transcriptional regulator | DNA/RNA metabolism | B |
| TWO78289.1 | ABC transporter ATP-binding protein | Membrane transport & Secretion | B |
| TWO78293.1 | undecaprenyl-diphosphooligosaccharide--protein glycotransferase | Metabolism | B |
| TWO78319.1 | oligoendopeptidase F | Other / Unknown | B |
| TWO78323.1 | apolipoprotein N-acyltransferase | Cell envelope / Periplasm | B |
| TWO78363.1 | UDP-N-acetylmuramate--L-alanine ligase | DNA/RNA metabolism | B |
| TWO78397.1 | ABC transporter substrate-binding protein | Membrane transport & Secretion | B |
| TWO78400.1 | ABC transporter ATP-binding protein | Membrane transport & Secretion | B |
| TWO78405.1 | potassium transporter TrkA | Membrane transport & Secretion | B |
| TWO78458.1 | N-carbamoylputrescine amidohydrolase | Other / Unknown | B |
| TWO78474.1 | argininosuccinate lyase | Metabolism | B |
| TWO78475.1 | redox-regulated ATPase YchF | Other / Unknown | B |
| TWO78487.1 | ribose-phosphate pyrophosphokinase | Metabolism | B |
| TWO78488.1 | carbon starvation protein A | Other / Unknown | B |
| TWO78496.1 | copper chaperone PCu(A)C | Stress response | B |
| TWO78502.1 | amino acid ABC transporter ATP-binding protein | Membrane transport & Secretion | B |
| TWO78553.1 | class 1 fructose-bisphosphatase | Other / Unknown | B |
| TWO78565.1 | threonine ammonia-lyase | Metabolism | B |
| TWO78591.1 | DUF342 domain-containing protein | Other / Unknown | B |
| TWO78617.1 | methionine ABC transporter ATP-binding protein | Membrane transport & Secretion | B |

**Suplementary Table S2:** Proteins were predicted using the EffectiveT3 server. Functional network associations were inferred from STRING v12 interaction analysis and are reported as descriptive modules rather than direct secretion evidence.

| **Protein ID** | **Protein annotation** | **EffectiveT3 score** | **Confidence level** | **Secretion** | **STRING-associated functional network** |
| --- | --- | --- | --- | --- | --- |
| TWO76580.1 | Hypothetical protein E6O49_02525 | 1.000 | High | True | Uncharacterized; weak STRING connectivity |
| TWO77279.1 | Ribonuclease J | 1.000 | High | True | RNA metabolism / ribosome-associated module |
| TWO77325.1 | Radical SAM/SPASM domain-containing protein | 1.000 | High | True | Cofactor biosynthesis / redox-associated cluster |
| TWO77761.1 | Multidrug efflux transporter outer membrane subunit CmeC | 1.000 | High | True | **CmeABC efflux system cluster** (CmeA/B/R) |
| TWO77854.1 | Prohibitin family protein | 1.000 | High | True | Membrane organization / stress-associated proteins |
| TWO78303.1 | ATP-dependent zinc metalloprotease FtsH | 1.000 | High | True | Membrane protein quality control module |
| TWO78332.1 | Polymer-forming cytoskeletal family protein | 1.000 | High | True | Cytoskeletal-like / cell shape maintenance |
| sp\|P13645\|K1C10_HUMAN | Keratin, type I cytoskeletal 10 (human) | 1.000 | High | True | **Host protein (serum contaminant)**; excluded from bacterial networks |
| TWO77943.1 | Glutamyl-tRNA reductase | 1.000 | High | True | Heme biosynthesis pathway |
| TWO77865.1 | Hydrogenase accessory protein HypB | 1.000 | High | True | [NiFe]-hydrogenase maturation module |
| TWO78028.1 | 50S ribosomal protein L28 | 1.000 | High | True | Ribosomal protein cluster |
| TWO77416.1 | DNA-directed RNA polymerase subunit alpha | 1.000 | High | True | Transcription machinery module |
| TWO77675.1 | Acetyl-CoA carboxylase carboxyltransferase subunit beta | 0.9998 | High | True | Fatty acid biosynthesis |
| TWO77252.1 | Citrate synthase | 0.9990 | High | True | Central carbon metabolism (TCA cycle) |
| TWO77321.1 | FAD-dependent thymidylate synthase | 0.9990 | High | True | DNA synthesis / nucleotide metabolism |
| TWO77942.1 | Proline--tRNA ligase | 0.9990 | High | True | Aminoacyl-tRNA synthetase module |
| TWO77111.1 | Aspartate--tRNA ligase | 0.9990 | High | True | Aminoacyl-tRNA synthetase module |
| TWO77194.1 | Iron-sulfur cluster assembly scaffold protein NifU | 0.9999 | High | True | Fe-S cluster biogenesis |
| TWO77654.1 | F0F1 ATP synthase subunit gamma | 0.9999 | High | True | Oxidative phosphorylation |
| TWO77628.1 | Cytolethal distending toxin nuclease subunit CdtB | 0.9999 | High | True | **CDT toxin complex (CdtA/B/C)** |
| TWO75454.1 | Flagellar hook protein FlgE | 0.9994 | High | True | **Flagellar assembly / T3SS-associated module** |
| TWO77295.1 | Flagellar hook protein FlgE | 0.9994 | High | True | **Flagellar assembly / T3SS-associated module** |
| TWO78535.1 | UDP-N-acetylglucosamine 1-carboxyvinyltransferase | 0.9986 | High | True | Peptidoglycan biosynthesis |
| TWO77568.1 | Acetate--CoA ligase | 0.9973 | High | True | Acetate metabolism |
| TWO76322.1 | META domain-containing protein | 0.9942 | High | True | Uncharacterized metabolic-associated cluster |
| TWO77434.1 | HugZ family heme oxygenase | 0.9940 | High | True | Heme utilization / iron metabolism |
| TWO77270.1 | 30S ribosomal protein S3 | 0.9917 | High | True | Ribosomal protein cluster |
| TWO75382.1 | Periplasmic nitratereductase subunit alpha | 0.9744 | Medium–High | True | Anaerobic respiration |
| TWO77963.1 | Molecular chaperone HtpG | 0.9739 | Medium–High | True | Stress response / protein folding |
| TWO78096.1 | Phosphate acetyltransferase | 0.9712 | Medium–High | True | Acetyl-phosphate metabolism |
| TWO78612.1 | Thiol peroxidase | 0.9556 | Medium–High | True | Oxidative stress defense |
| TWO77230.1 | Trigger factor | 0.9524 | Medium–High | True | Ribosome-associated protein folding |
